# Multi-Functional and Multi-Scale Aspects in Polymer Composites

**DOI:** 10.3390/polym16243475

**Published:** 2024-12-13

**Authors:** Md Najib Alam, Vineet Kumar

**Affiliations:** School of Mechanical Engineering, Yeungnam University, 280, Daehak-ro, Gyeongsan 38541, Republic of Korea; mdnajib.alam3@gmail.com

## 1. Introduction

Polymer composites have gained attention due to their multifunctionality and ability to tune properties at multiple scales. These polymer composites contain filler particles at the macro, micro, and nanoscale, reinforcing polymer matrices [1]. This polymer matrix can be thermoplastic, thermoset, or elastomer. Among them, composites based on the elastomer matrix are more promising due to their ability to stretch, their easy processing, and their lightweight nature. Finally, these composite materials offer a unique combination of properties that can be tailored to meet specific application requirements [2]. The emerging focus on multi-functional and multi-scale aspects has revolutionized their design and broadened their applicability. They are tailoring the polymer composite for a particular application such as obtaining composites with different properties [3]. These properties are mechanical stiffness, different thermal conductivity, or desired electrical conductivity. Moreover, the multi-scale approach in polymer composites involves tuning the composite material structures across multiple length scales—nano, micro, and macro—to achieve desired properties. This strategy helps in combining the benefits of small-scale fillers and large-scale architectures [4].

In the present Special Issue, the term “multifunctional” refers to composites’ ability to deliver more than one performance attribute. Common applications of these composite materials include aerospace, flexible electronics, sensors, and self-powered generators [5]. Moreover, their light weight is critical to help tailor properties. The multi-scale design approach ensures optimal material performance by leveraging nano-reinforcements, hierarchical structuring, and controlled matrix–filler interactions [6]. Moreover, the term “multi-scale” refers to considering material structure and behavior across different scales. For example, (a) the macroscale is used for achieving structural integrity and load-bearing capacity, (b) the microscale is used for distribution and orientation of filler particles, and (c) the nanoscale refers to interfacial interactions and surface chemistry dependency for the overall properties of polymer composites [7,8]. The multi-scale approach allows the aspects related to simulation and modeling at multiple scales to help predict properties and performance, reducing development costs. Moreover, combining nano-fillers with micro-reinforcements can create unique hierarchical structures that improve performances synergistically [9]. In the end, optimizing the interface between the matrix and fillers ensures effective stress transfer and minimizes defects at the nano and microscales. Keeping these aspects in mind, the present Special Issue focuses on the interplay between multi-functional and multi-scale aspects in polymer composites. These aspects offer a powerful toolkit for engineering advanced materials for tuning properties and applications of interest. The coming section will summarize the articles published in this Special Issue.

## 2. Overview of Published Articles

Torres et al. [10] report eco-friendly composites based on natural rubber and jute for the footwear industry. The results show that the treated jute fiber sample exhibits a lower hardness of up to 8% and higher tensile strength of 70% compared to the control sample. Moreover, at 10 phr, the crosslinking density was ~3.5 × 10^−4^ mol/cm^3^ for the untreated jute fiber and increased to ~9.5 × 10^−4^ mol/cm^3^ for the treated jute fiber-based composites. The authors further report that the abrasion resistance was 58% for the untreated jute fiber and 68.3% for the treated jute fiber-based composites. Thus, a higher abrasion resistance was found for the treated jute fiber. In another study by Shchegolkov et al. [11], composites based on organosilicon elastomers and metallic microspheres, MWCNTs, were fabricated. The results show that the power at −40 °C was 1.1 kW/m^2^ and decreased to 0.3 kW/m^2^ after 100 cycles. However, with the addition of Ni additives, the power at −40 °C increased to 1.4 kW/m^2^ and then decreased to 1.2 kW/m^2^ after 100 cycles. Moreover, with the addition to Cu, the power at −40 °C increased to 1.6 kW/m^2^ and then decreased to 1.5 kW/m^2^ after 100 cycles. These results are quite promising for strain sensing and self-powered nanogenerators. Grygier et al. [12] investigate plastic manufacturing through the SLA-type additive method. The mechanical property tests show that the tensile strength was highest for the ABS-like sample, optimum for DLP craftsman resin, and lowest for the UV-sensitive resin sample. For example, it was 21.42 MPa (ABS-like sample), 12.86 MPa (DLP craftsman resin), and 0.01 MPa (UV-sensitive resin). In another research activity by Luo et al. [13], electrically conductive fibers were fabricated from composites based on natural rubber, and MXene was reported. These composites were finally tested for strain-sensing applications and promising results were reported. For example, the electrical conductivity was 10^−4^ S/cm at 1 wt% of MXene and it further increased to 100 S/cm at 5 wt% of MXene in natural rubber composites. However, the elongation at break decreased from 1400% at the unfilled sample to 200% at 20 wt% of MXene-filled natural rubber composites. Finally, the modulus was 0.8 MPa for an unfilled sample and increased to 100 MPa at 20 wt% of MXene in natural rubber composites. Lastly, Kumar et al. [14] report a review article on silicone rubber-based composites for multifunctional sensing systems. This review provides a summary of sensing performances reported in various research papers from 2019 to 2024. After describing the fabrication methodology, the mechanical and electromechanical properties are reported. The electromechanical properties are further described in terms of the response time, gauge factor, and finally the linearity of different composites. The final real-time applications of sensing for different types of composites were overviewed and reported. Finally, the conclusions, followed by the key challenges and future prospects of the work, are reported.

## 3. Challenges and Future Outlook

Polymer composites are engineered materials whose properties are dependent upon reinforcement offered by filler particles to the polymer matrix. However, there are various challenges in achieving high performance for the multi-functionalities of these innovative composites [15]. Some of these challenges are (a) achieving uniform dispersion of nanofillers in the polymer matrix; (b) achieving high polymer–filler compatibility and thus achieving high interfacial interaction; and (c) scaling high-performance and cost-effective composites [16]. The future of polymer composites in terms of multi-functional and multi-scale simulations is quite bright. The use of advanced materials in fabricating polymer composites is promising due to their self-sensing, self-healing, and shape-memory capabilities [17]. Additionally, the use of advanced manufacturing methods like 3D printing and automatic fiber dispersion can assist in achieving the desired properties. Moreover, the functionalization of new-generation fillers or the polymer matrix can simulate polymer–filler compatibility, thereby improving filler dispersion, and high interfacial bonding at the interface is achieved [18]. Further, AI and machine learning can be incorporated for predictive modeling across scales to design more efficient composites, for example for real-time monitoring of properties during processing to ensure optimal performance [19]. The increasing focus on sustainability and recyclable composites, biodegradable matrices, and renewable fillers offers a minimized environmental impact. Finally, applications in flexible electronics, wearable devices, and next-generation aerospace materials will expand as these composites become more accessible to society [20]. Overall, polymer composites are set to transform multiple industries, driven by advances in multi-functional and multi-scale innovations that bridge performance, sustainability, and manufacturing challenges.
